# Effect of increased convective clearance by on-line hemodiafiltration on all cause and cardiovascular mortality in chronic hemodialysis patients – the Dutch CONvective TRAnsport STudy (CONTRAST): rationale and design of a randomised controlled trial [ISRCTN38365125]

**DOI:** 10.1186/1468-6708-6-8

**Published:** 2005-05-20

**Authors:** E Lars Penne, Peter J Blankestijn, Michiel L Bots, Marinus A van den Dorpel, Muriel P Grooteman, Menso J Nubé, Ingeborg van der Tweel, Piet M ter Wee, the CONTRAST study group

**Affiliations:** 1 Department of Nephrology, University Medical Center Utrecht, Heidelberglaan 100, 3584 CX Utrecht, The Netherlands; 2 Julius Center for Health Sciences and Primary Care, University Medical Center Utrecht, Universiteitsweg 100, 3584 CG Utrecht, The Netherlands; 3 Department of Internal Medicine, Rijnmond-Zuid Medical Center, Clara Location, Olympiaweg 350, 3078 HT Rotterdam, The Netherlands; 4 Department of Nephrology, VU Medical Center, De Boelelaan 1117, 1081 HV Amsterdam, The Netherlands; 5 Department of Internal Medicine, Medical Center Alkmaar, Wilhelminalaan 12, 1815 JD Alkmaar, The Netherlands; 6 Center for Biostatistics, Utrecht University, Padualaan 14, 3584 CH Utrecht, The Netherlands

**Keywords:** End stage renal disease, hemodialysis, hemodiafiltration, convective transport, middle molecules, mortality, cardiovascular disease, outcome

## Abstract

**Background:**

The high incidence of cardiovascular disease in patients with end stage renal disease (ESRD) is related to the accumulation of uremic toxins in the middle and large-middle molecular weight range. As online hemodiafiltration (HDF) removes these molecules more effectively than standard hemodialysis (HD), it has been suggested that online HDF improves survival and cardiovascular outcome. Thus far, no conclusive data of HDF on target organ damage and cardiovascular morbidity and mortality are available. Therefore, the CONvective TRAnsport STudy (CONTRAST) has been initiated.

**Methods:**

CONTRAST is a Dutch multi-center randomised controlled trial. In this trial, approximately 800 chronic hemodialysis patients will be randomised between online HDF and low-flux HD, and followed for three years. The primary endpoint is all cause mortality. The main secondary outcome variables are fatal and non-fatal cardiovascular events.

**Conclusion:**

The study is designed to provide conclusive evidence whether online HDF leads to a lower mortality and less cardiovascular events as compared to standard HD.

## Background and rationale

Atherosclerotic cardiovascular disease (CVD) is common among hemodialysis (HD) patients. In fact, approximately 50% of the deaths is attributed to cardiovascular causes, which is much higher than in the general population [1]. In addition, chronic HD patients suffer from atherosclerotic complications at a relatively younger age and die younger from ischemic heart disease [2]. The origin of CVD in chronic HD patients is most probably multi-factorial, as the extremely high prevalence in this patient group is not easily explained by traditional risk factors, either alone or in combination [3]. In recent years, other contributing factors have emerged, including the accumulation of uremic toxins, disturbances in the immuno-inflammatory system, as reflected by a chronic micro-inflammatory state, increased oxidative stress, and endothelial dysfunction [4-6]. In particular the retention of larger uremic toxins, the so-called middle molecules (MM, molecular weight [MW] 0.5 – 50 kDa), may play an important role in the pathogenesis of CVD [7,8]. Therefore, it is conceivable that dialysis modalities with superior MM removal reduce CVD and improve survival.

In contrast to diffusive dialysis strategies, which mainly remove small MW solutes, such as urea and creatinine, convective dialysis strategies are particularly effective in the removal of larger molecules. In hemodiafiltration (HDF), diffusive and convective transport are combined, providing an optimal removal of both small and larger MW substances up to the range of 30 – 40 kDa. Clinical studies have shown that beta-2-microglobulin (β2 m), which is a typical MM with a MW of 11.8 kDa and therefore incapable of passage through the membrane of low flux devices, is effectively removed during HDF leading to lower pre-dialysis levels in the long term [9,10]. Similarly, the removal of other MM such as advanced glycation end-products (AGEs), leptin, and complement factor D is enhanced by convective transport [11-13]. Apart from the increased MM clearance, it has been suggested that HDF improves the removal of smaller molecules that are highly protein bound, due to a better elimination of the unbound fraction [14]. With respect to homocysteine, which is >90% protein bound, the observed decrease may also be explained by an improved removal of uremic substances with inhibitory effects on its metabolism [15]. Finally, it has been shown that treatment with online HDF leads to lower plasma phosphate concentrations, as compared to standard HD [16,17].

At present, it is unclear whether HDF has a favourable effect on the micro-inflammatory state in dialysis patients. Although a reduction of pro-inflammatory proteins has been shown during HDF [18], anti-inflammatory cytokines may also be removed. Of note, besides solute removal, other factors may influence the inflammatory-state as well, such as the bio-incompatibility of the dialyser membrane and the microbiological quality of the dialysate [19,20]. Considering oxidative stress and endothelial dysfunction, data on the effects of HDF on these parameters are limited.

**Figure 1:**
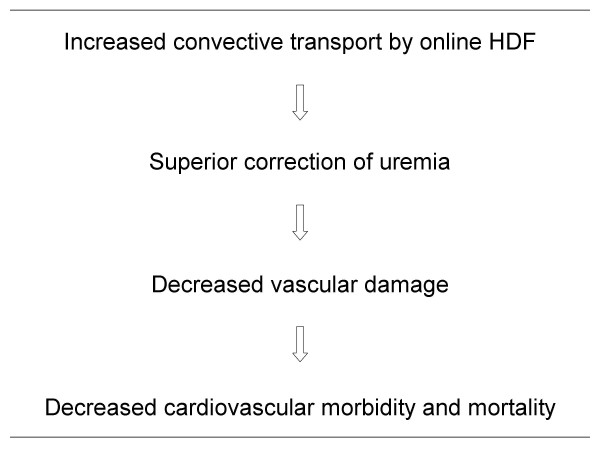
Hypothesis

In summary, compared to standard HD, HDF improves the uremic state by an increased clearance of MM and other, mainly protein bound, uremic toxins. Circumstantial evidence implies that these effects result in less vascular damage and ultimately in decreased cardiovascular morbidity and mortality (figure 1). Although observational studies suggest that online HDF improves cardiovascular outcome in chronic HD patients [21,22], two small randomised studies failed to show any differences between online HDF and standard HD [23,24]. However, the latter analysis lacked adequate power to detect differences in clinical endpoints.

Based on the above-mentioned theoretical considerations, the scarcity of reliable clinical data, and the growing interest in convective techniques under nephrologists, the CONvective TRAnsport STudy (CONTRAST) has been initiated. CONTRAST is a randomised controlled trial investigating the effects of online HDF on clinical endpoints, compared to low-flux HD. If online HDF indeed leads to an improvement in CV morbidity and mortality, this finding will imply a breakthrough in the treatment of chronic HD patients.

## Methods

### Objectives

The primary objective of CONTRAST is to assess the effect of on-line HDF on all cause mortality, when compared to standard low-flux HD. The main secondary outcomes are fatal and non-fatal cardiovascular events. Other secondary outcome measures include differences between treatment regimens on the progression of left ventricular mass index (LVMi), as assessed by echocardiography, the progression of atherosclerosis as assessed by measurement of carotid intima-media thickness (CIMT) and the progression of arterial stiffness, as assessed by measurement of aortic pulse wave velocity (PWV). Furthermore, several laboratory markers of endothelial function, inflammatory state, and oxidative stress will be assessed over time and compared between the two treatment groups. In addition, subjective global assessment (SGA) is performed in the study patients as a measure of nutritional state, and a questionnaire is used to investigate the effects of on-line HDF on quality of life.

### Study design

In this randomised controlled trial, participants are randomised centrally into a 1:1 ratio for treatment with online HDF or treatment with low-flux HD. Randomisation is stratified by the participating centres and occurs in blocks. The follow-up period is three years. At present, 24 dialysis centres have agreed to recruit the required number of patients. The study is conducted according to good clinical practice (GCP) guidelines.

#### Patients

The in- and exclusion criteria are given in table 1. Since the study results may be of importance for chronic HD patients of all ages, no upper age limit has been set. Severe incompliance is defined as non-adherence to the dialysis prescription, especially the frequency and duration of dialysis treatment. Permission for participation in other (e.g. observational) studies will be discussed with and decided by the executive committee.

**Table 1:** Inclusion and exclusion criteria

**Inclusion criteria**
patients treated by HD 2 or 3 times a week, for at least 2 months.
patients able to understand the study procedures.
patients willing to provide written informed consent.


**Exclusion criteria**

current age < 18 years
treatment by HDF or high flux HD in the preceding 6 months
severe incompliance
life expectancy < 3 months due to non renal disease
participation to other clinical intervention trials evaluating cardiovascular outcome

#### Stabilisation period

Before randomisation, patients will be dialysed 3 times (or 2 times) per week with low-flux synthetic dialysers (UF-coefficient < 20 ml/mmHg/h) for at least 6 months in case of a prevalent dialysis patient and at least 2 months in case of a new dialysis patient.

Blood flow will be maintained at 250–400 ml/min. Anticoagulation is performed with low molecular weight heparin (LMWH) before HD. Patients on coumarins receive 50% of the LMWH dose. Treatment times will be adapted to a target dialysis spKt/V urea of ≥ 1.2 per treatment. Ultra pure water is used for preparation of dialysis fluid. Bicarbonate is provided from powder cartridges to avoid the risk of a bacterial load from bicarbonate concentrates. For instance, the biBAG^R ^system (Fresenius) and BiCart^R ^system (Gambro) will be used. The dialysate flow is 500 ml/min and the temperature of the dialysate is 36°C.

#### Routine patient care

Metabolic control will be performed according to the guidelines of the Quality of Care Committee of the Dutch Federation of Nephrology. Anti-hypertensive medication, lipid lowering therapy, platelet aggregation inhibitors and medication to treat renal anemia and renal osteodystrophy will also be prescribed according to these guidelines, and, if not available, according to usual care.

#### Randomisation

The patients will be randomised as soon as they are considered to be stable. When a patient has been randomised for low-flux HD, the treatment as performed in the stabilisation period will be continued. Treatment times will be adjusted only if dialysis spKt/V urea < 1.2 per treatment or if ultrafiltration goals can not be achieved, according to the attending nephrologist. When randomised for online HDF, patients will be treated with a target post-dilution dose of 6 l/h (~100 ml/min) and a high-flux synthetic dialyser (UF-coefficient > 20 ml/mmHg/h). Blood flow will be set at >300 ml/min, if possible, in order to achieve a substitution volume of 100 ml/min. If the blood flow is less than 300 ml/min, the post-dilution volume will be decreased accordingly (filtration and post-dilution <25–33% of blood flow). If necessary, the dose of LMWH will be increased and given in two separate doses. Treatment times will be fixed according to the prescription in the stabilisation period and adjusted only when spKt/V urea is < 1.2 / treatment. Metabolic control and medication is similar to the low-flux group, as described above.

#### Dialyser membranes

Dialysers with comparable biocompatible membrane material and surface area will be used in both treatment groups, to ascertain that differences in clearance result from differences in convective transport, rather than differences in dialyser characteristics. Only if the target dose of 6 l/h post-dilution is not achieved in the online HDF patients, it is allowed to prescribe a membrane with a larger surface area. The membranes advised by the study group are summarised in table 2. As many low-flux membranes with a membrane surface > 1.5 m^2 ^have a UF coefficient 10–20 ml/mmHg/h, in this study low-flux is defined as a UF coefficient of < 20 ml/mmHg/h.

**Table 2:** Dialyser characteristics for both treatment arms

	**Low-flux HD**		**Online HDF**	
**Company**	Gambro	Fresenius	Gambro	Fresenius
**Dialyser**	Polyflux 17L	F8HPS	Polyflux 170H	FX80
**Membrane material**	polyamide	polysulfone	polyamide	polysulfone (helixone)
**Sterilisation method**	heat	heat	heat	heat
**Surface area (m^2 ^)**	1.7	1.8	1.7	1.8
**Membrane thickness (μm)**	50	40	50	35
**UF coefficient (ml/mmHg/h)**	13	18	65	59
**In vitro clearance:**^# ^				
**Urea**	260	251	268	276
**Phosphate**	198	193	229	239
**Vit B12**	111	118	158	175

# (QB = 300 ml/min, QD = 500 ml/min)

#### Online HDF technique

During hemodiafiltration, the removal of larger solutes is increased by excess ultrafiltration, leading to solute removal by convection. As fluid removal exceeds the desired weight loss of the patient, fluid balance is maintained by the infusion of a pyrogen-free substitution solution. In addition, dialysate is used to create a concentration gradient for solute removal by diffusion, as in standard HD. At the introduction of HDF more than 20 years ago, the substitution fluid was supplied in bags. The infusion volumes were limited due to the high costs and laborious procedure, limiting the efficiency of HDF.

In recent years, however, technical advances have made it possible to prepare the substitution solution online from ultra pure water and dialysate concentrates. As a result, the volume of the substitution fluid could be increased considerably, without the disadvantages of inconvenient bags. Hence, the UF rate can be increased up to 50L per treatment in the pre-dilution mode and 25L in the post-dilution mode [25].

#### Online dialysate and substitution fluid preparation

Ultra-pure water is used for the preparation of bicarbonate-containing dialysis fluid, which undergoes one step of ultrafiltration converting it into ultra pure dialysis fluid. Dialysis fluid is produced at a rate of 600–800 ml/min of which approximately 100 ml/min is diverted for further processing into substitution fluid. The electrolyte composition of the dialysis fluid is: Na^+ ^138–140 mmol/l; K^+ ^1.0–3.0 mmol/l; HCO_3 _^- ^30–35 mmol/l; Ca^++ ^1.0–1.7 mmol/l; Mg^++ ^0.5 mmol/l; Cl^- ^108–109.5 mmol/l; glucose 0–5.6 mmol/l; acetate 3 mmol/l.

The substitution fluid is prepared from the dialysis fluid by one additional step of controlled ultrafiltration, before it is infused post-filter into the blood. The electrolyte composition of the substitution fluid is the same as the composition of the dialysis fluid. Ultrafiltration procedures will be performed according to the manufacturers' instructions, as described below.

- The on-line system, ONLINEplus™ (Fresenius Medical Care, Bad Homburg, Germany) is integrated into the dialysis machine (4008 series; Fresenius Medical Care) and consists of two ultrafilters (DIASAFE^® ^plus), an infusate pump module, and disposable infusate lines. Infusate is prepared continuously by double-stage ultrafiltration. Both filters are subjected to automated membrane integrity tests before dialysis, and are replaced after 100 treatments or 12 weeks of use, whichever comes first. Dialysis fluid downstream from the first filter stage enters the dialyser; part of the stream is subjected to cross-flow filtration in the second filter in order to produce infusate. The infusate stream is connected with the venous bubble catcher for post-dilution HDF [25,26].

- The AK 100/200 ULTRA dialysis machine (Gambro AB, Lund, Sweden) prepares ultra pure water and ultra pure dialysis fluid by stepwise ultrafiltration of water and bicarbonate -containing dialysis fluid (BiCart) using two polyamide ultrafilters (U8000 S). When used for HDF, sterile non-pyrogenic solution is prepared on-line from the ultra pure dialysis fluid by an additional step of ultrafiltration using a sterile polyamide ultrafilter (U2000) integrated in a sterile line set (Steriset). The hygiene of the fluid pathway, including the U8000S ultrafilters, will be assured by heat disinfections after each treatment. The U8000S filters are changed bimonthly. The final ultrafilter (U2000) is employed on a single-use basis [26,25].

### Data collection

#### Baseline and follow-up data registration

At baseline, all relevant information will be documented: i.e. demographical data, information on cardiovascular risk factors, time on dialysis, cause of renal insufficiency, and medication. A follow-up visit will be scheduled every three months. During this visit, the occurrence of CV events, death, and hospitalisation will be documented. In addition, blood pressure, body weight and the achieved filtration and substitution dose per treatment will be registered. Case record forms are provided using the TeleForm system (version 8.1.1, Cardiff Software Inc, Vista, CA, USA). As all completed forms are scanned, no data entry by typing is needed. Registration of all data will be performed in each centre by the attending nephrologists and research nurses.

#### Recording outcome events

CV events include fatal or non-fatal myocardial infarction, stroke, therapeutic coronary procedure (PTCA or stenting), therapeutic carotid procedure (endarterectomy or stenting), and PTA and vascular intervention (revascularisation, PTA or stenting). Congestive heart failure is excluded as a CV event, since the discrimination with fluid overload is often hard to make in chronic HD patients. Furthermore, hospitalisations, duration of the hospitalisations and main diagnosis (including the occurrence of infections) will be recorded during the study period.

An independent event committee will evaluate all causes of death, cardiovascular events, and infections. The primary investigators will collect sufficient information of the events for the event committee. The event committee is blinded for information on the received treatment and consists of physicians with different specialisations: neurologists, vascular surgeons, nephrologists, internists, and cardiologists. Events will be coded as fatal and non-fatal, definite, probable and possible and not codeable (i.e. insufficient information). Only definite and probable events will be used in the final analysis. This procedure is successfully applied in a number of studies coordinated by the Julius Center, e.g. in the SMART study [27].

#### Left ventricular hypertrophy

Using transthoracic M-mode echocardiography from the parasternal long axis position, left ventricular end-diastolic diameter (LVEDD), end-systolic diameter (LVESD) as well as posterior and septal wall thickness will be determined at baseline, after 6 months, after 12 months and annually afterwards, on a midweek non-dialysis day according to a central uniform protocol. From these parameters left ventricular ejection fraction (LVEF) will be determined as LVEDD – LVESD/LVEDD, while the left ventricular mass index (LVMi) will be calculated using the formula of Devereux and Reichek [28], modified in accordance with the recommendations of the American Society of Echocardiography [29]. The ultrasound investigations will be recorded on a compact disc and analysed off-line by experienced cardiologists in a core laboratory.

#### Vessel wall measurements

With respect to carotid intima-media thickness (CIMT), the outcome is the change in mean common CIMT, defined as the average of the intima-media thickness measurements performed circumferentially at pre-defined angles for the near and far wall of 10 mm segments of the right and left distal common carotid arteries [30]. A limited number of centres will be involved in the CIMT measurements in this study. Centres will be trained according to a central uniform carotid ultrasound protocol. Before actually starting the study, sonographers need to be certified as outlined in the CIMT ultrasound protocol.

Measurements will be performed at baseline and then annually on a midweek non-dialysis day. The ultrasound scan is being recorded on videotape and analysed off line by a core laboratory. Quality Assurance and Quality Control procedures as existing and applied in several (inter)national trials will be implemented [31,32].

Pulse wave velocity (PWV) is determined to provide additional information on functional changes of the arterial wall [33]. The outcome measurement is the change in aortic PWV. A limited number of centres will be involved in the PWV measurements in this study. Centres will be trained according to a central uniform PWV protocol. Data are checked regularly on quality control aspects as defined in the PWV protocol as described earlier [34].

Measurements will be performed at baseline and then annually on a midweek non-dialysis day.

#### Nutritional state

At base-line, after 1, 2 years and at the end of the study, nutritional state is assessed by subjective global assessment (SGA), pre-albumin and dry weight [35].

#### Quality of life

Patient well-being will be estimated at baseline, and once a year by the Kidney Disease Quality of Life Short Form (KDQOL-SF). This version is validated in American and Dutch dialysis patients [36,37].

#### Laboratory assessments

Three monthly, blood samples will be drawn for routine laboratory assessments. In addition, blood samples will be taken at baseline, and after 6, 12, 18, 24, and 36 months for determinations of oxidative stress, inflammatory and endothelial function markers. Finally, a whole blood sample will be stored for future research on the effect of genetics on the response to HDF, after specific permission of the patients in the informed consent form.

### Statistical methods

The results of the study will be analysed according to the 'intention to treat' principle.

#### Primary outcome

The primary outcome variable is the time until the occurrence of an event defined as 'all cause mortality'. Results will be presented as Kaplan-Meier curves for the two treatment arms and the difference between the two treatments will be analysed using a log-rank test. The log-rank test will be adjusted for the effect of cumulative data analyses (see below).

#### Secondary outcomes

CV events are considered as secondary outcome variables. They will be analysed and presented as described for the primary outcome variable.

The primary analysis of CIMT progression will employ a linear random coefficient (Laird-Ware) model using real visit days, treatment and clinical center as independent variables. for each participant, the intercept and slope of CIMT change over time is assumed to be a normally distributed random variable with different means for the two treatment groups. The mean slope for the HDF treatment group will be compared to that for the low flux group using linear contrasts and a 5% significance level. Additional exploratory analyses will evaluate the impact of including baseline CIMT, lumen diameter, and ultrasound reader as additional co-variates.

The data analytic approach to arrive at the PWV outcome variable and the LVMi outcome variable is similar to that of the CIMT outcome. Adjustments that will be taken into account in the estimates are changes in MAP and changes in heart rate, since both are closely related to PWV.

#### Sample size considerations

The sample size of the present study is based on the following event rates: the 3-year all cause mortality rate among subjects with ESRD is 44% based on data from the Dutch renal replacement registry (RENINE) [38]. CV mortality constitutes 40–60% of the total group of deaths, leading to a 3 year CV mortality rate of 22% in HD patients. Assuming that the incidence of non-fatal CVD is equal to the CV mortality rate (22%), the three-year incidence of fatal and non-fatal CVD is 44%. In addition, based on experience ± 8% of the ESRD patients will undergo renal transplantation yearly and as such is being censored in the trial.

Assuming that HDF will reduce all cause mortality with 20%, it has been estimated that with a two-sided alpha of 0.05 and a power of 80%, about 772 patients need to be enrolled and followed for three years. In these patients about 250 events are expected to come to a decision. Note that the total number of patients to be included cannot be specified in advance because of the planned sequential interim analyses, as described below.

#### Interim analysis

In this study, group sequential interim analyses will be performed to evaluate the primary outcome variable. The reason for this approach is that, on average, fewer patients are needed in the study when the expected difference in the primary outcome variable is real or when no difference of the hypothesised magnitude can be expected anymore.

Sequential analysis is a statistical approach where one conducts significance tests over time as the data are collected. Sequential analysis and its application in clinical trials have been described extensively by Whitehead [39]. Sequential design and analysis is implemented in the computer program PEST version 4 [40].

**Figure 2:**
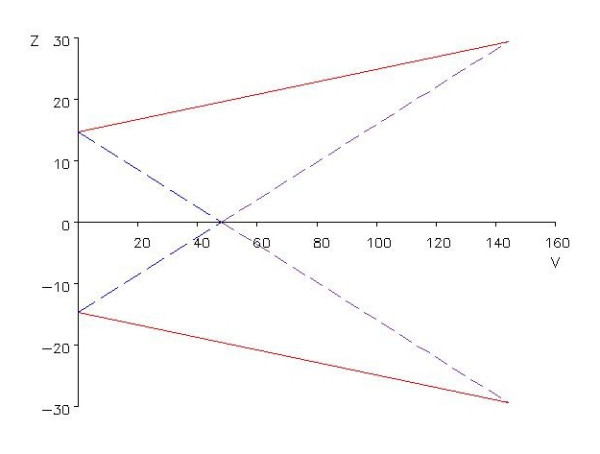
**Sequential analysis**. Boundaries for a double sequential triangular test with α = 0.05, power 0.80 and hazard ratio 0.75. Z is the observed number of events in the control group minus the expected number of events given treatment equivalence. V is approximately equal to a quarter of the number of events observed.

The general approach is as follows. A null hypothesis H_0 _and an alternative hypothesis H_1 _are formulated for a suitable measure θ of treatment difference. For this study with a survival type outcome variable, θ is equal to the negative of the logarithm of the hazard ratio (HR). The HR is defined as the ratio of the logarithm of the (expected) cumulative survival under HDF (= 0.648) and the logarithm of the (expected) cumulative survival under HD (= 0.56). H_0 _is formulated as "no difference in the occurrence of the primary endpoint between the two trial arms" or θ = 0 (i.e. HR = 1). The alternative hypothesis H_1 _is formulated as |θ| ≥ -log(0.75) = 0.29. Two test statistics, Z and V, can be derived depending on the type of response variable. Z is a measure of the treatment difference; for survival data Z is the observed number of events in the control group minus the expected number of events given treatment equivalence. V reflects the amount of information about θ contained in Z; for survival data V is approximately equal to a quarter of the number of events observed. The sequential analysis requires critical boundaries to be specified in advance. These boundaries depend on θ, the type I error α and the power 1-β. For each new group of patients, values of Z and V are calculated and presented graphically by plotting Z against V (see Fig. 2 for an illustration of a double-sided sequential test). Based on the path of cumulative (Z,V)-points, one of the following three decisions is made:

1) the upper or the lower (continuous) boundary is crossed: stop the data collection and reject the null hypothesis;

2) one of the inner wedge-shaped (dashed) boundaries is crossed: stop the data collection and accept the null hypothesis;

3) continue the data collection: the cumulative data are inadequate to draw a conclusion yet.

An independent Data Safety and Monitoring Board (DSMB) will evaluate the results of the sequential interim analyses. The DSMB consists of a biostatistician (chair), a nephrologist, an internist, and a clinical epidemiologist. The biostatistician will perform the sequential analyses. The executive committee will provide the DSMB every 2 months with the relevant database to perform the unblinded analyses. The main task of the DSMB is to decide whether the analyses provide enough evidence of either efficacy or no efficacy with respect to the primary outcome and formulates recommendations for the executive committee on the continuation of the trial. The DSMB may also offer unsolicited recommendations on the continuation of the trial, for example after publication of results of similar trials.

## Conclusion

Online HDF is gaining popularity, as recent technical advances have made it possible to safely replace considerable amounts of fluid at reasonable cost. In addition, accumulating evidence indicates that the correction of the uremic state is improved by online HDF, if compared to standard HD. However, at present it is unclear whether long-term treatment with HDF ultimately results in an improved clinical outcome. Therefore, CONTRAST is initiated, a randomised controlled trial of sufficient sample size to detect differences in survival and cardiovascular events. Patients will be randomised between low-flux HD and online HDF and followed for 3 years. Over 20 Dutch dialysis centers participate in this study and approximately 800 incident and prevalent HD patients will be recruited. By April 2005, more than 150 patients were included.

## Appendix

### Steering committee

The steering committee consists of the primary investigators (nephrologists) of the participating centers. The members and institutions in the Netherlands are:

W. Bax, Medical Center Alkmaar, Alkmaar;

W.H. Boer, University Medical Center Utrecht, Utrecht;

H. Boom, Reinier de Graaf Hospital, Delft;

G.J. Bruinings, Slingeland Hospital, Doetinchem;

M. van Buren, Leyenburg Hospital, The Hague;

G.W. Feith, Gelderse Vallei Hospital, Ede;

A.B. Geers, St Antonius Hospital, Nieuwegein;

J.O. Groeneveld, Onze Lieve Vrouwe Gasthuis, Amsterdam;

H.W. van Hamersvelt, University Medical Center St Radboud, Nijmegen;

F. de Heer, Maasland Hospital, Sittard;

B.C. van Jaarsveld, Dianet Dialysis Centers, Utrecht;

M.G. Koopman, Academic Medical Center, Amsterdam;

A.T. Lavrijssen, Oosterschelde Hospital, Goes;

C.J. Konings, Catharina Hospital, Eindhoven;

M.I. Koolen, Jeroen Bosch Hospital, 's Hertogenbosch;

T.K. Kremer Hovinga, Martini Hospital, Groningen;

W.H. van Kuijk, VieCuri Medical Center, Venlo;

J.J. Offerman, Isala Clinics, Zwolle;

L.J. Reichert, Rijnstate Hospital, Arnhem;

P.L. Rensma, St Elisabeth Hospital, Tilburg;

C.E. Siegert, Sint Lucas Andreas Hospital, Amsterdam;

P.J. van de Ven, Rijnmond-Zuid Medical Center, Rotterdam;

M.G. Vervloet, VU Medical Center, Amsterdam.

In Norway:

H.J. Kloke, Haukeland Hospital, Bergen.

### Executive committee

P.J. Blankestijn, nephrologist, University Medical Center Utrecht, Utrecht (chair);

M.L. Bots, epidemiologist, University Medical Center Utrecht, Utrecht;

M.A. van den Dorpel, nephrologist, Rijnmond-Zuid Medical Center, Rotterdam;

M.P. Grooteman, nephrologist, VU Medical Center, Amsterdam;

M.J. Nubé, nephrologist, Medical Center Alkmaar, Alkmaar;

E.L. Penne, research physician, University Medical Center Utrecht, Utrecht;

P.M. ter Wee, nephrologist, VU Medical Center, Amsterdam (chair).

### Data Safety and Monitoring Board

D.E. Grobbee, epidemiologist, University Medical Center Utrecht, Utrecht;

A.J. Rabelink, nephrologist, Leiden University Medical Center, Leiden;

C.D. Stehouwer, internist, Academic Hospital Maastricht, Maastricht;

I. van der Tweel, biostatistician, Center for Biostatistics, Utrecht University, Utrecht.

### Event committee

P.A. Doevendans, cardiologist, University Medical Center Utrecht, Utrecht;

L.J. Kapelle, neurologist, University Medical Center Utrecht, Utrecht;

G. Ligtenberg, nephrologist, Utrecht;

F. Stam, internist, VU Medical Center, Amsterdam;

G. Veen, cardiologist, VU Medical Center, Amsterdam;

M.C. Visser, neurologist, VU Medical Center, Amsterdam;

F.L. Visseren, internist, University Medical Center Utrecht, Utrecht;

W. Wisselink, vascular surgeon, VU Medical Center, Amsterdam.

### Advisory committee

P. Boer, biochemist, University Medical Center Utrecht, Utrecht;

M.J. Cramer, cardiologist, University Medical Center Utrecht, Utrecht;

O. Kamp, cardiologist, VU Medical Center, Amsterdam;

B. van Rossum, cardiologist, VU Medical Center, Amsterdam;

C. Schalkwijk, biochemist, VU Medical Center, Amsterdam;

C.D. Stehouwer, internist, Academic Hospital Maastricht, Maastricht;

T. Teerlink, biochemist, VU Medical Center, Amsterdam.

### Trial coordinating centre

M.L. Bots, epidemiologist, University Medical Center Utrecht, Utrecht;

L. ten Brinke, CRA, Hans Mak Institute, Naarden;

M.P. Grooteman, nephrologist, VU Medical Center, Amsterdam;

E.A. Ram, project manager, University Medical Center Utrecht, Utrecht.

Project management and data management is provided by the Julius Center for Health Sciences and Primary Care, University Medical Center Utrecht, Utrecht.

## Competing interests

The author(s) declare that they have no competing interests.

## Authors' contributions

ELP has drafted the manuscript and has contributed to the design. PJB, MLB, MAD, MPG, MJN and PMW have designed the trial and have been involved in revising the article. IT has made contributions to the study design and has drafted the statistical methods section of this manuscript. All authors have approved the final manuscript.
